# Gradient Annealing as a New Strategy to Fabricate Gradient Nanoparticle Array on Microwires

**DOI:** 10.1186/s11671-022-03698-0

**Published:** 2022-06-20

**Authors:** Anqi Chen, You Lv, Yanyan Wu, Yuan Zhu

**Affiliations:** 1 School of Microelectronics, Southern University of Science and Technology, Shenzhen, 518055 China; 2 School of Innovation and Entrepreneurship, Southern University of Science and Technology, Shenzhen, 518055 China

**Keywords:** Microwire, Doping gradient, Temperature gradient, Gradient nanoparticle array, Surface enhanced Raman scattering

## Abstract

Creating gradients of nanostructure on the surface has found broad applications such as enhanced optical spectroscopy, optical storage of information, and broadband solar energy harvesting. Here, a facile strategy is presented for fabricating gradient nanoparticle arrays with tunable size. It takes a ZnO:Ga microwire as the starting material, and the Ga^3+^ doping gradient along the microwire is induced by the high voltage applied. Such a doping gradient facilitates the formation of a temperature gradient in a Joule heating process. And this temperature gradient produced by this technique can be as high as 800 °C/mm, which could be later used for gradient annealing of thin metal films. After annealing, the thin metal films turn to gradient nanoparticle arrays. The obtained gradient nanoparticle arrays are confirmed effective in multi-wavelength surface enhanced Raman scattering enhancement.

**Supplementary Information:**

The online version contains supplementary material available at 10.1186/s11671-022-03698-0.

## Introduction

Over the past decade, there has been growing research interest in gradient materials, which display gradient properties such as feature sizes, chemistry composition, morphology, and phase structure [1–5]. Such materials are of great value in many applications due to their unique properties. On the one hand, gradient materials are envisaged to have new phenomena and properties associated with the gradually changing interfaces and multiscale hybridization [6]. On the other hand, material gradients can be designed and tailored as a “materials genome” that a single sample can be used to investigate the variable properties in a combinatorial manner. Thus, high-throughput screening and cost-effective property testing of materials are enabled [7].

One of the most widely studied configurations is substrate-supported gradient nanoparticle arrays (GNPA), either in random or ordered arrangements [7, 8]. Metal nanoparticle arrays with size/density gradient can introduce gradual change in optical response, scattering, and absorption, which are very useful in sensing [9], optoelectronics [10], and surface-enhanced Raman scattering (SERS) [11].

Two typical fabrication approaches to GNPA are (a) Top-down methods including electron-beam lithography [12], focused ion beam lithography [13], and (b) Bottom-up methods including chemical adsorption [7], dip-coating [8], and template assistance self-assembly [14]. The top-down methods are generally suffered from high cost and low yield, while the bottom-up methods cannot avoid the use of complex chemicals.

Here we demonstrate a facile gradient thermal annealing method to produce GNPA. The strategy is to induce a doping gradient along a semiconductor microwire and then build a temperature gradient by Joule heating to anneal thin metal films coated, which soon turn to GNPA. The resultant GNPA exhibited strong SERS responses to multi-wavelength excitations. The presented approach shows a cost-effective fabrication of gradient metal nanostructure and can be further used for multi-wavelength response SERS application.

## Experimental

### Microwire Growth and Devices Fabrication

Ga-doped ZnO microwires used in this letter were grown via the vapor–liquid–solid (VLS) growth method [15]. Briefly, high purity metallic zinc (3N) powder and gallium (3N) slug were mixed at a proper ratio (10:1) and filled into a half-open quartz tube, and then placed on the edge of a horizontal tube furnace. Then a quartz boat coated with 100 nm Au film was placed in the center of the tube furnace as the catalysis. The microwires with different diameters and lengths were obtained under the growth temperature of 960–1000 °C for 2 h in the open air (Additional file 1: Fig. S1a).

After growth, the single microwire was transferred from the quartz boat to a sapphire substrate by glass fiber. And two In electrodes were fixed at both ends of the individual ZnO:Ga microwire. Then the device was annealed for 1 min at 200 °C for better ohmic contact. The current versus voltage (I–V) measurement and the electrical heating experiment of the device were performed using Keysight B2902A system.

### Fabrication of SERS Substrate and Characterization

The ZnO:Ga microwire was coated with 10 nm thin gold film using electron beam evaporation. And the SERS substrate was created after Joule heating this microwire for 30 s. The surface morphology was characterized by field emission scanning electron microscopy (FESEM, Hitachi S4800), and the element mapping was carried out with the attached energy dispersive X-ray spectroscopy (EDX, Bruck Max 50). Temperature measurement was carried out by an optics IR camera. The resistance mapping of the microwire was carried out using an ultrafine probe mounted on precise high-precision 3-axis linear stages (Additional file 1: Fig. S2). For the SERS testing, R6G (99%, Aladdin) was solved in pure ethanol to obtain R6G/ethanol solution (with a concentration of 10^−6^). The SERS substrates were soaked in the corresponding solution for 1 h, then washed using pure ethanol and dried by a nitrogen gun. Raman spectra were collected by a HORIBA LabRAM HR Evolution system with 532 nm and 785 nm incident laser.

## Results and Discussion

**Fig. 1:**
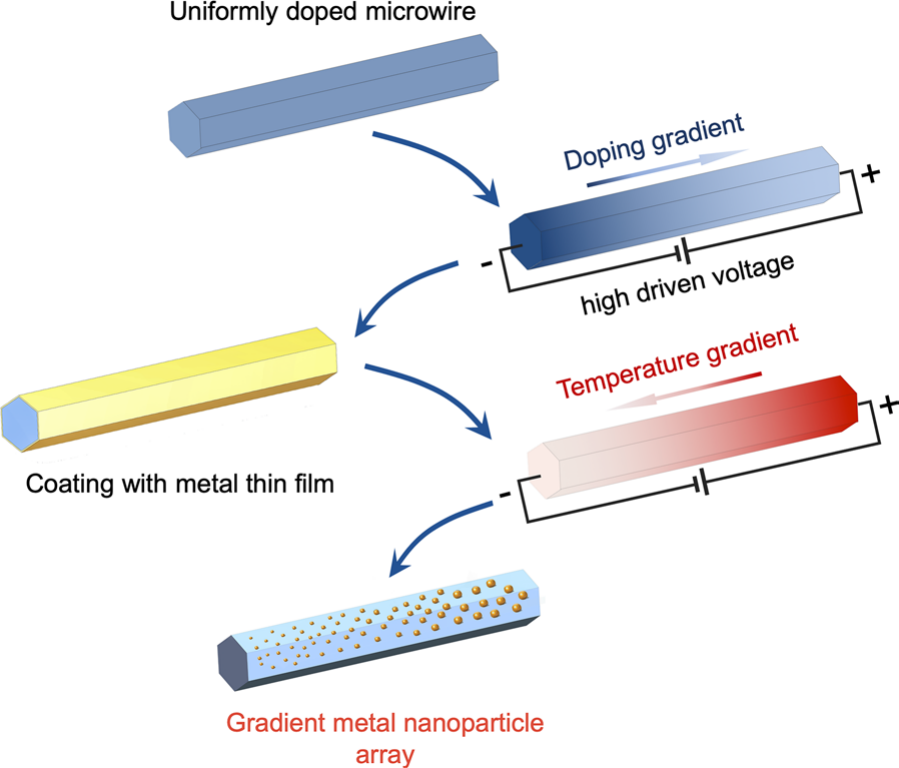
Fabrication scheme of metal GNPA

The fabrication strategy is based on an ingeniously designed temperature gradient for thin metal layer annealing, which is sketched in Fig. 1. Starting with a uniformly heavy-doped microwire, we apply high voltage on it to drive dopants transfer and thus obtain a certain resistivity gradient. After coating a thin layer of the metal film around the microwire, we apply a lower voltage to it. The Joule heating helps to build a temperature gradient, which renders a gradient annealing condition to the metal film. The dewetting performance of the metal film is dependent on the annealing temperature, which in this case is gradient. The annealing process is kept short (tens of seconds) to obtain the wanted GNPA.

### Build of Impurity Gradient

**Fig. 2:**
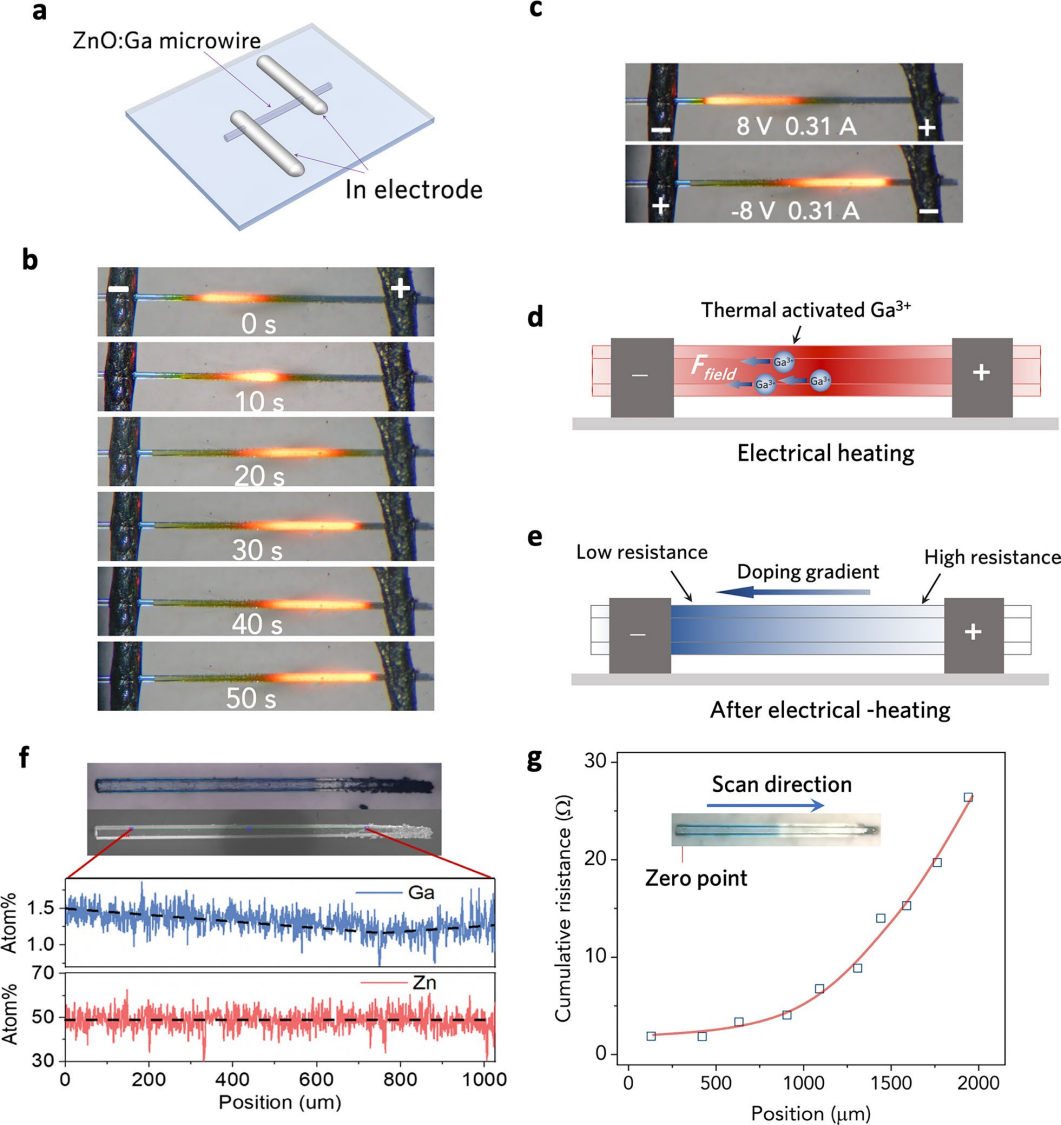
**a** Schematic of the Ga-doped microwire heating device. **b** The optical image of hotspot transport when the high voltage was applied. **c** The hotspot position under forward and reverse bias. **d** Schematic diagram of the impurity migration in the Joule heated microwire. **e** The regulated resistance along the microwire after impurity migration. **f** The optical/SEM image of the microwire after dopants redistribution and the EDS line scan spectrum along the microwire. **g** Line-scan of the resistance along the microwire

The highly Ga-doped microwire (~ 10^20^ cm^3^) was chosen to be the heating wire of the micro heating device due to its high electrical conductivity, the high melting temperature of 1975 °C [16], and high thermal stability in air. The as-grown ZnO:Ga microwire shows a uniform dark blue color (Additional file 1: Fig. S1c), indicating that the dopant concentration was uniform lengthwise. To fabricate the microwire heating device, the microwire was transferred onto the two Indium electrodes (with 1.5 mm spacing) via micro-manipulation and then annealed at 200 °C for better Ohmic contact (Fig. 2a). The purpose of this arrangement is to create a small gap between the microwire and the substrate and reduce heat dissipation through the substrate.

At the beginning, the optical image (Additional file 1: Fig. S3) shows uniform temperature distribution along the microwire as a low voltage (5 V, ~ 2.5 kA/cm^2^) was applied. However, when a high voltage was applied to the microwire (8 V, ~ 4 kA/cm^2^), we observed the hotspot gradually move from the negative electrode to the positive (Fig. 2b). We can also notice that the hot spot on the microwire always moves toward the lower potential end no matter under positive or reverse bias (Fig. 2c). This phenomenon was mainly attributed to the electromigration of Ga dopants, which is illustrated in Fig. 2d. When a high voltage (8 V) was applied to the microwire, the Ga dopants will be activated due to the Joule heating effect. Then the electrostatic force caused by the electric field will drive the Ga dopants to migrate from the positive side to the negative side. Microcosmically, during the doping, the Ga^3+^ (~ 0.62 Å) will occupy the host lattice of the Zn^2+^ (~ 0.74 Å). Owing to the difference regarding the ion radius and chemical states, the dopant of Ga^3+^ presents less stability than the host Zn^2+^. Within a lattice framework, the less stable ion will migrate as the ion was driven by an external force (such as electric field or high temperature). In that case, a minimum migration barrier height is demanded to be overcome. According to our repeated experiments, we deem that the migration barrier height is larger than 5 V and smaller than 8 V. Therefore, only a larger driving force applied (~ 8 V, both electric field force and thermal driving force here) could overcome such a potential barrier, while ~ 5 V is used for subsequent heating experiment. Such impurity electromigration phenomenon can be also found in other II–VI semiconductors, such as CdS and ZnSe [17]. When Ga dopants migrated to the left, the resistance of the right side will increase, thus leading to the movement of the hotspot. Finally, a dopant density gradient was created along this microwire (Fig. 2e). We can also notice that the color of the right side of the microwire becomes light after electrical heating, which indicates that the dopants have been redistributed under the electric field. The EDS line scan result further verified the Ga impurity gradient along the microwire (Fig. 2f). Furthermore, we characterized the resistance distribution of the microwire using a micro probing system. The measurement was carried out by fixing a probe at the left end of the microwire and line-scanning the resistance along the axial direction of the microwire using another probe, which is illustrated in Additional file 1: Fig. S2. The relation between the cumulative resistance and the distance of the two probes (Fig. 2g) indicates that the resistivity at the different positions of the microwire increases from the left end to the right end (Additional file 1: Fig. S4). In other words, a resistivity gradient was created along the microwire.

### Build of Temperature Gradient

**Fig. 3:**
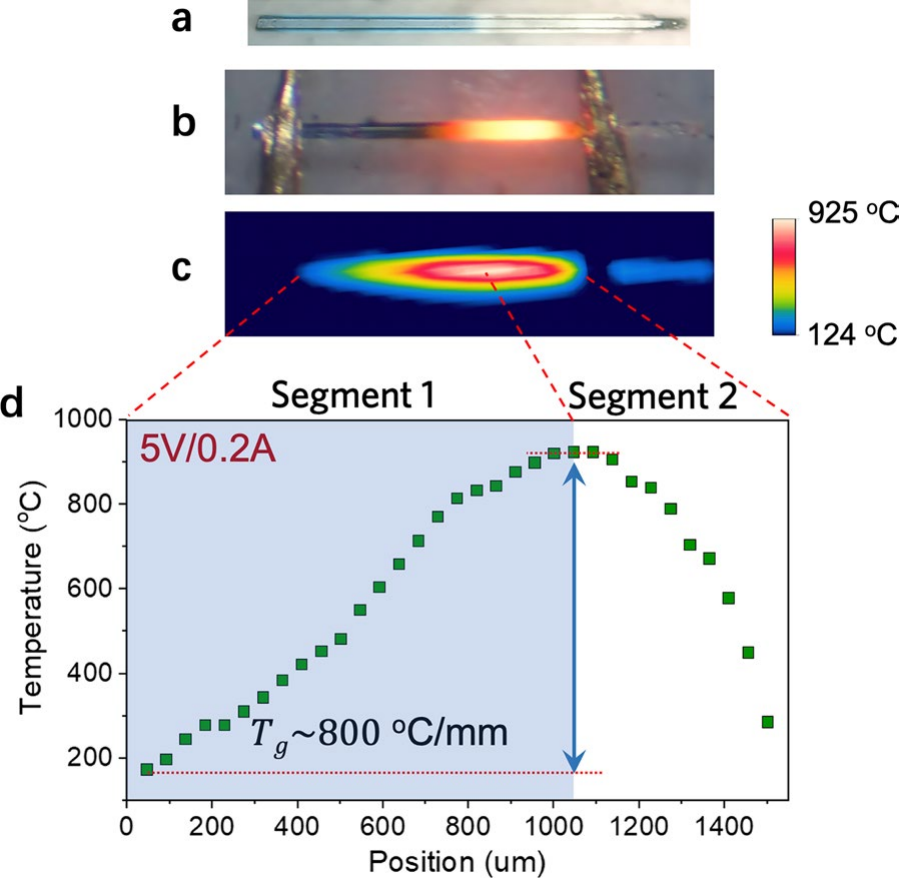
**a** Optical image of the microwire with doping gradient. **b** Optical image and **c** IR image of the microwire under Joule heating. **d** The temperature distribution along this microwire

Since the Joule heat is proportional to the resistance of the microwire, the heating effect is expected to be more significant on the high resistance side. Therefore, after the resistivity gradient was created (Fig. 3a), a lower voltage was applied to this device (5 V, ~ 2.5 kA/cm^2^). The hotspot was shifted to the right side of the microwire where the resistivity is higher (Fig. 3b). The IR image in Fig. 3c also verified the shift in the hot spot location. The temperature on the hotspot reached more than 900 °C, while the temperature near the left electrode pad was below 200 °C. The corresponding one-dimensional temperature profile along the Joule-heated microwire is shown in Fig. 3d. Due to the inhomogeneous heating effect, a temperature gradient as high as 800 °C/mm was created along the microwire marked with segment 1. The temperature gradient is quite stable even after the heating wire is powered on for tens of minutes.

### Fabrication of Metal GNPA

**Fig. 4:**
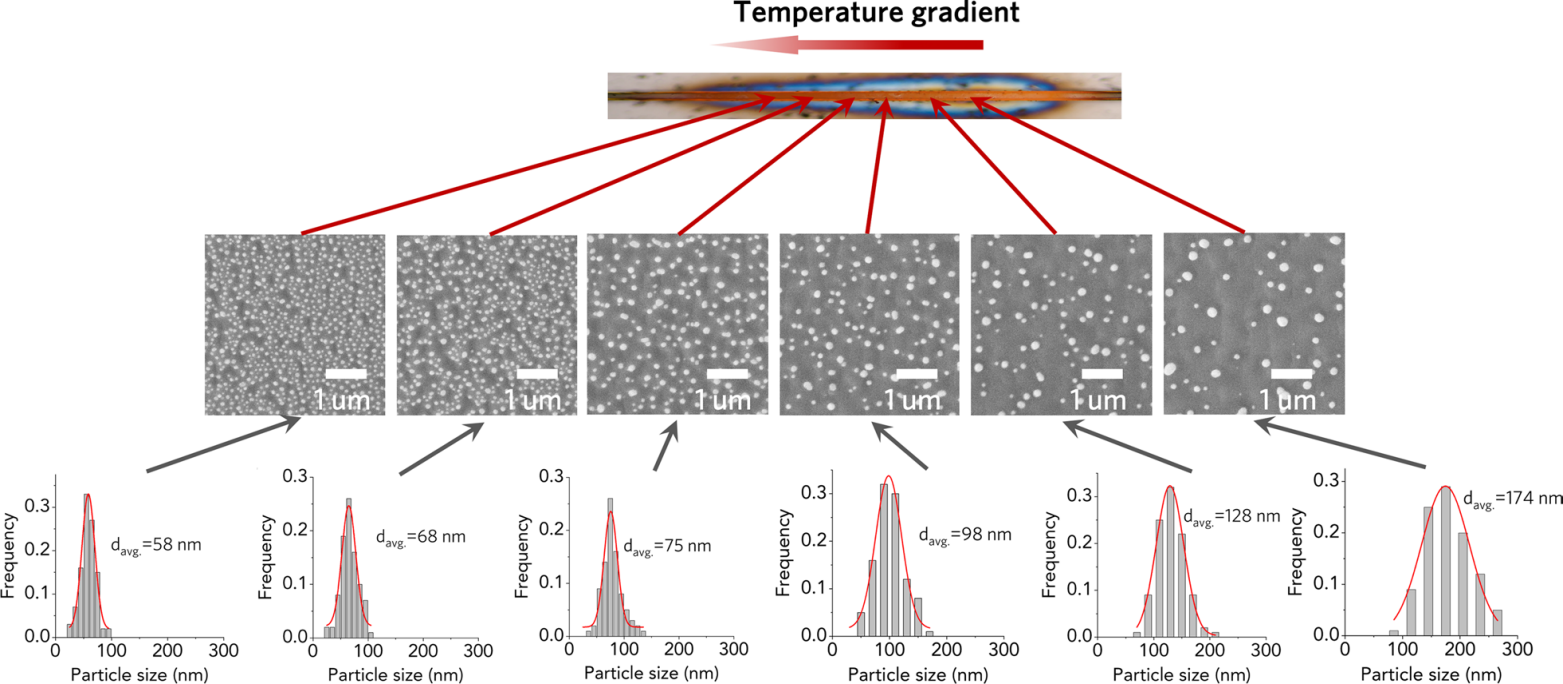
SEM images of different positions along the microwire after thermal gradient annealing and corresponding size distributions of particles in each image

The fabrication of metal GNPA is based on temperature-dependent layer dewetting, including the following steps: (1) A thin layer (10 nm) of the gold film was coated on the microwire device by evaporation; (2) The micro heating device was switched on. The thin film was soon melted and coalesced into the nanosphere by surface tension; (3) The micro heating device was switched off. It is known that the average size of the metal nanoparticle is strongly dependent on the annealing temperature [18, 19]. Based on this behavior, gold nanoparticle arrays with a size gradient can be fabricated, which can be identified from the SEM images at each position of the heating wire (Fig. 4). The corresponding average particle sizes are also calculated, which are shown below. The average size of the gold nanoparticle increases from ~ 58 nm in the region with a lower temperature (~ 500 °C) to ~ 174 nm in the region with a higher temperature (~ 800 °C). The above results prove that the gold GNPA can be readily created by applying an appropriate thermal gradient in the annealing process. It is also noted that this strategy is quite universal and can be extended to other metals, such as platinum (Additional file 1: Fig. S5).

### GNPA in Multi-wavelength Response SERS Application

**Fig. 5:**
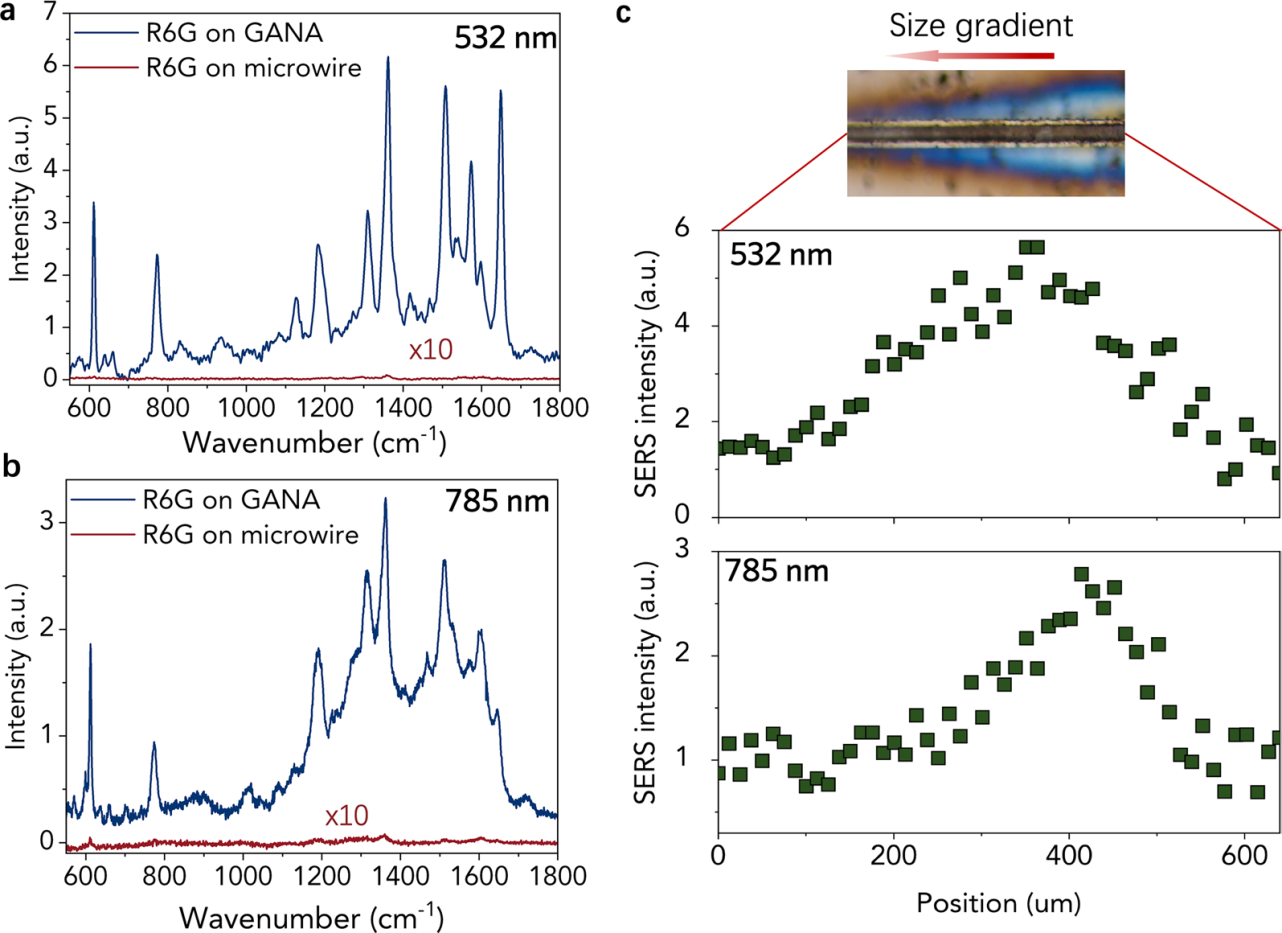
Typical SERS spectra of R6G on microwire with and without gold GNPA at **a** 532 nm and **b** 785 nm laser excitation. **c** SERS line scan along the size gradient shows the variation of the position-dependent SERS intensity

Nanoparticle arrays with size gradient can introduce gradual change in resonance peak position, which is especially useful for acting as multi-wavelength response SERS substrates. To demonstrate such application, microwires with and without gold GNPA were soaked in the R6G/ethanol solution with the concentration of 10^−6^ m for the absorption of R6G molecules, which are as probe molecules to study the enhancement performance of SERS substrates. And excitation laser lines at 532 nm and 785 nm were employed to test the SERS enhancement of gold GNPA. The typical surface-enhanced Raman spectra of R6G at 532 nm and 785 nm laser excitation are shown in Fig. 5a, b. It exhibits distinct Raman modes of R6G (612, 772, 1308, 1361, 1505, 1575, and 1649 cm^−1^) for the microwire with gold GNPA under both 532 nm and 785 nm laser excitation**.** All the Raman bands of R6G are significantly enhanced compared with the microwire without gold GNPA. SERS line mapping along the size gradient of the gold GNPA was also carried out. Under the 532 nm laser excitation, the SERS signal of the R6G increased firstly and then decreased, and the maximum intensity occurred in the position of 350 μm. This result can be attributed to the fact that localized surface plasmon resonances (LSPR) are tightly related to the gold nanoparticle size. It is clear that there is a proper particle size for gold nanoarrays to realize a maximum Raman scattering enhancement. When the excitation wavelength was switched to 785 nm, the SERS line mapping curve shows similar trends. However, the maximum signal slightly shifts to the position of 420 μm where the nanoparticle size is larger. Normally, the resonance peak redshifts with increasing nanoparticle size [20, 21]. Thus, when the wavelength of the excitation laser increases, the maximum enhancement shift to the position with a larger nanoparticle size. We also calculated the averaged surface enhancement factor of the gold GNPA SERS substrate (see detailed calculation in Additional file 1) [20]. The maximum enhancement factors under 532 nm and 785 nm laser excitation are 5.4 × 10^4^ and 1.7 × 10^4^. The above findings prove that our gold GAPA produces strong SERS responses to multi-wavelength excitation lines, which could provide a one-chip high-efficiency solution for multi-wavelength SERS detection.

## Conclusion

In conclusion, we have presented a facile strategy for the formation of gradient metal nanoparticles array with continuous particle size. We started from Ga-doped ZnO microwire and created a doping gradient along the microwire by applying a high voltage on it. We found that such a doping gradient can facilitate the formation of a stable temperature gradient (~ 800 °C/mm) along the microwire in a Joule heating process. After annealing a thin metal film coated on this microwire using this temperature gradient, GNPA can be produced. The resultant GNPA exhibited a gradual change in plasmonic properties and induced a multi-wavelength SERS response in the presence of molecular analytes. The strategy presented here is quite universal and can be also extended to other metals.

## Supplementary Information

**Additional file 1: Fig. S1.** Material growth and characterization of Ga-doped ZnO microwire. (a) Growth set-up of the Ga-doped ZnO microwire. (b) SEM image and (c) optical image of the Ga-doped ZnO microwire. **Fig. S2.** Schematic diagram of testing set-up for line scan of the cumulative resistance alone the microwire. **Fig. S3.** The optical image of microwire when the lower voltage was applied at the beginning. **Fig. S4.** First derivative of cumulative resistance to position. **Fig. S5.** SEM images of the Pt nanoparticles at different positions along the microwire after thermal gradient annealing. Calculation of the enhancement factor.

## Data Availability

The data used and analyzed during the current study are available from the corresponding authors upon reasonable request.

## Abbreviations

GNPA: Gradient nanoparticle arrays
SERS: Surface-enhanced Raman scattering
VLS: Vapor–liquid–solid
FESEM: Field emission scanning electron microscopy
EDX: Energy dispersive X-ray spectroscopy
LSPR: Localized surface plasmon resonances
