# Supplementary material for: Gradient Annealing as a New Strategy to Fabricate Gradient Nanoparticle Array on Microwires
Source: Nanoscale Res Lett. 2022 Jun 20;17:59. doi: 10.1186/s11671-022-03698-0 (PMC9209622; doi:10.1186/s11671-022-03698-0)
Supplement: Supplementary file 1 — Additional file 1: Fig. S1. Material growth and characterization of Ga-doped ZnO microwire. (a) Growth set-up of the Ga-doped ZnO microwire. (b) SEM image and (c) optical image of the Ga-doped ZnO microwire. Fig. S2. Schematic diagram of testing set-up for line scan of the cumulative resistance alone the microwire. Fig. S3. The optical image of microwire when the lower voltage was applied at the beginning. Fig. S4. First derivative of cumulative resistance to position. Fig. S5. SEM images of the Pt nanoparticles at different positions along the microwire after thermal gradient annealing. Calculation of the enhancement factor. [file 11671_2022_3698_MOESM1_ESM.docx]

**Supporting Information**

**Gradient annealing as a new strategy to fabricate gradient nanoparticle array**


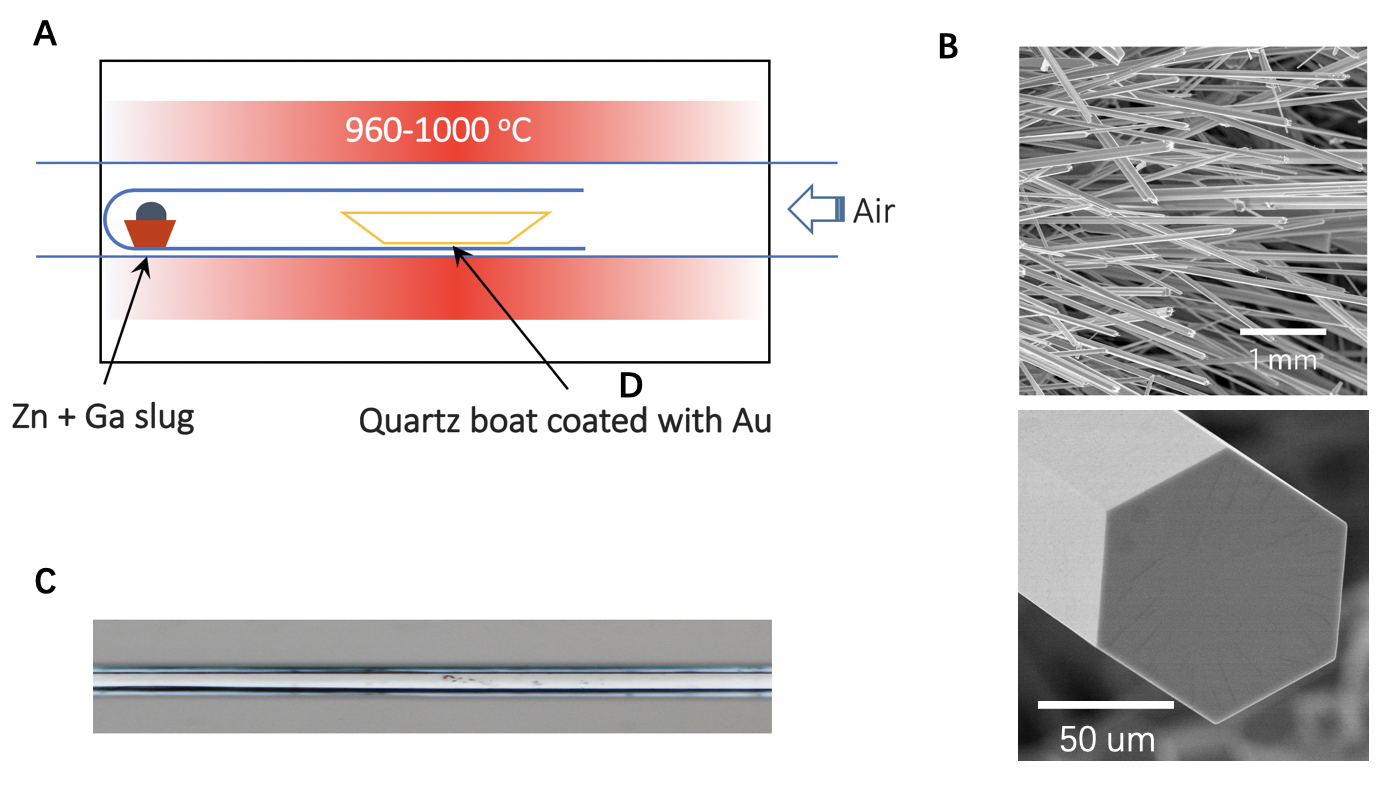


**Fig. S1** Material growth and characterization of Ga doped ZnO microwire. (a) Growth set-up of the Ga doped ZnO microwire. (b) SEM image and (c) optical image of the Ga doped ZnO microwire.


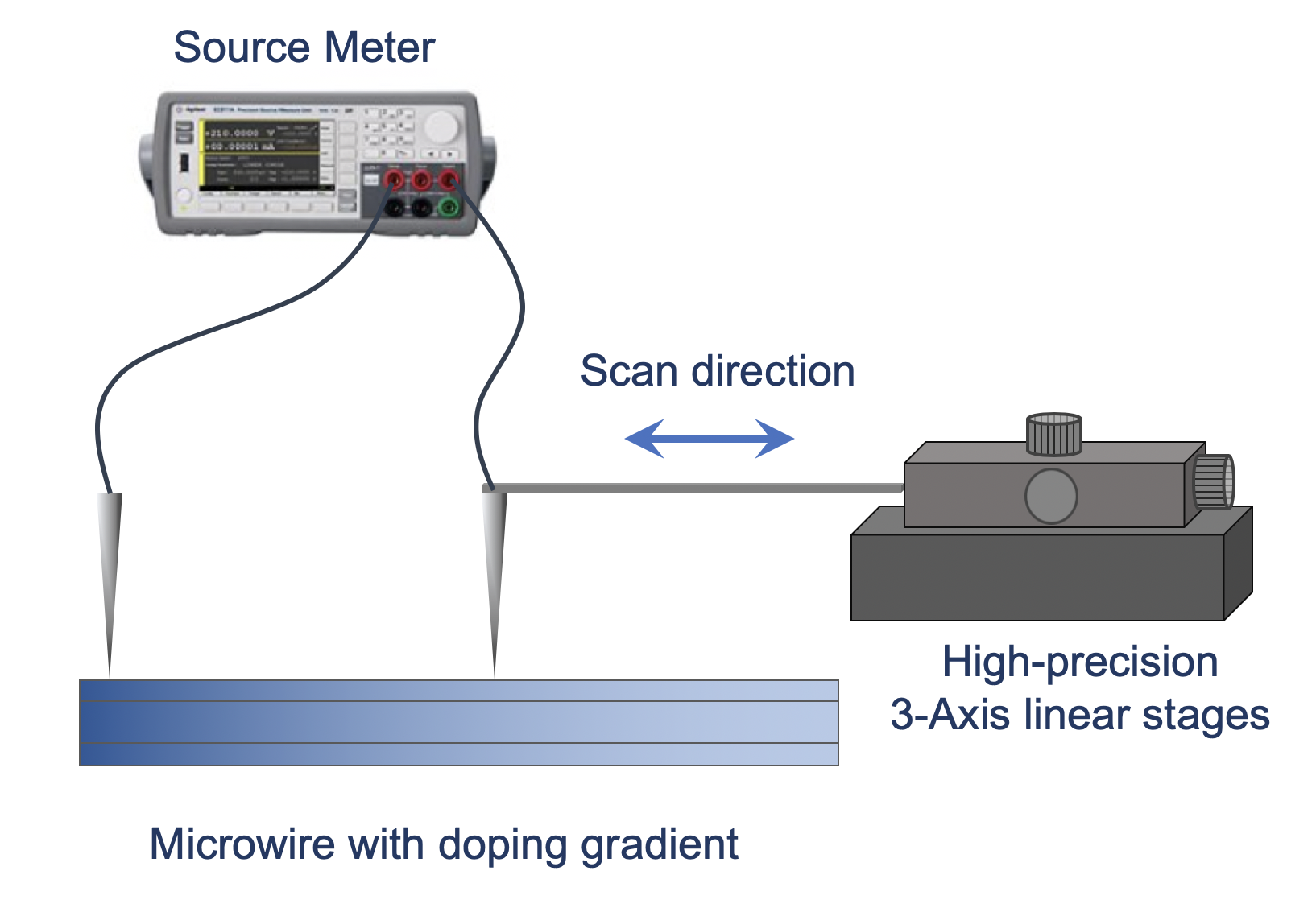


**Fig. S2** Schematic diagram of testing set-up for line scan of the cumulative resistance alone the microwire.


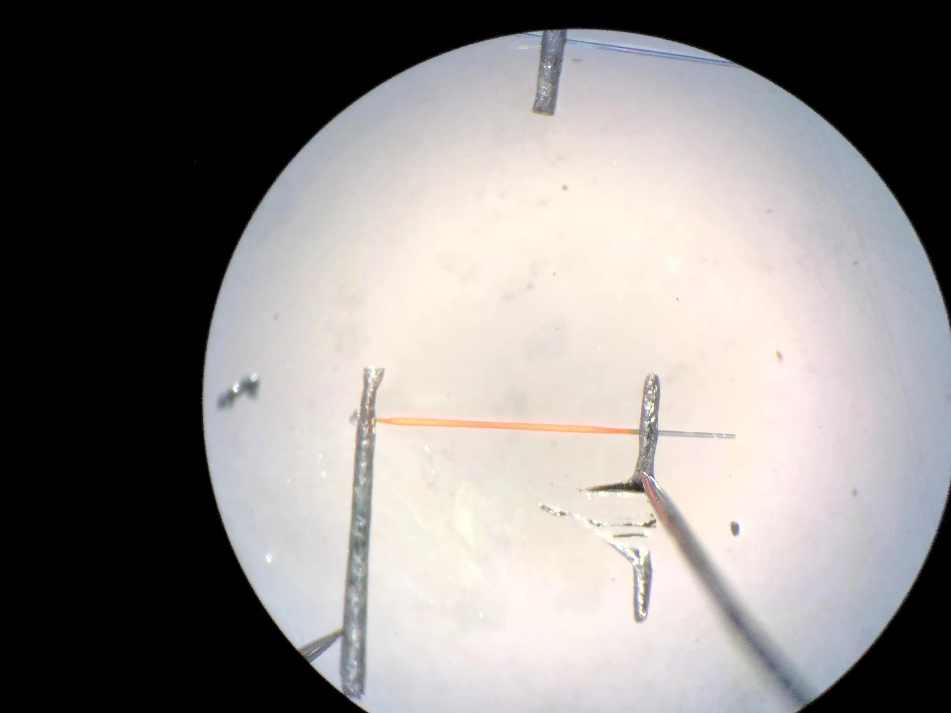


**Fig. S3** The optical image of microwire when the lower voltage was applied at the beginning.

**Fig. S4** 1st derivative of cumulative resistance to position.

the relationships between cumulative resistance and resistivity per unit length can approximately be written by:

| $R \left( x \right)=\int_{0}^{x} \rho\left( x \right)dx$ | (1) |
| --- | --- |
| $\rho\left( x \right)=\frac{dR}{dx}$ | (2) |

Where *x* is the distance from zero point, *R* is the cumulative resistance, *ρ* is the resistivity per unit length. So the 1st derivative of cumulative resistance in Fig. S3 can be regarded as the resistivity per unit length as well.


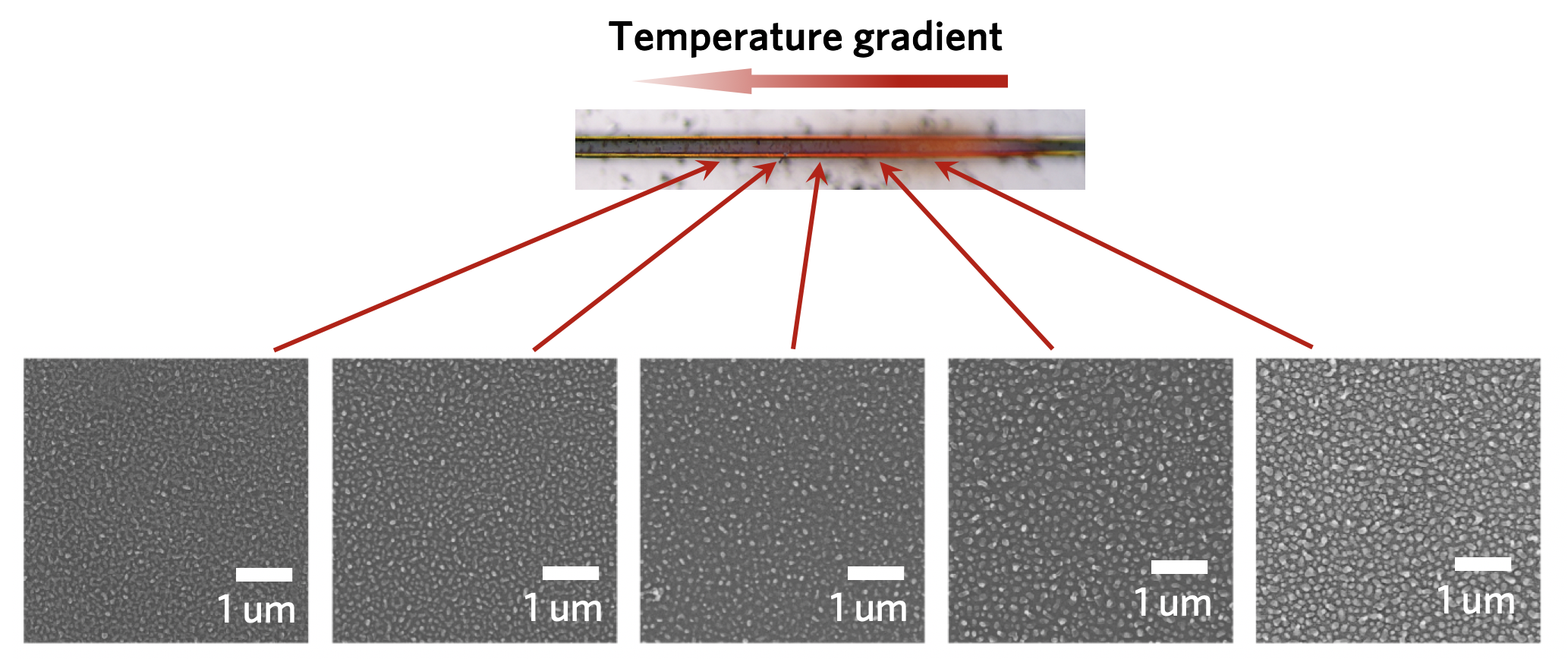


**Fig. S5** SEM images of the Pt nanoparticles at different positions along the microwire after thermal gradient annealing.

**Calculation of the enhancement factor:**

The enhancement factor of the gold GNPA SERS substrate is based on the following equation: [1]

$$EF=\frac{I_{SERS}}{I_{normal}}\cdot\frac{cN_{A}\sigma h}{R}$$

Where *I_SERS_* and *I_normal_* are the intensity of Raman signal of the SERS spectroscopy and normal Raman spectroscopy, respectively, *c* is the concentration of R6G solution for normal Raman test (10^-2^ mol/L in our experiment), N_A_ is the Avogadro constant, *σ* is the occupied area of a single R6G molecule (1.65 nm^2^), *R* is the height to width ratio of gold nanoparticle (~1), *h* is the thickness of the ultrathin layer of solution on glass substrate(~50 μm). The maximum enhancement factors under 532 nm and 785 nm laser excitation are 5.4×10^4^ and 1.7×10^4^.

**Reference**

1. Lin X M, Cui Y, Xu Y H, et al. (2009). Surface-enhanced Raman spectroscopy: substrate-related issues[J]. Analytical and bioanalytical chemistry, 394(7): 1729-1745.
